# The Disturbance of Hepatic and Serous Lipids in Aristolochic Acid Ι Induced Rats for Hepatotoxicity Using Lipidomics Approach

**DOI:** 10.3390/molecules24203745

**Published:** 2019-10-17

**Authors:** Junyi Zhou, Yifei Yang, Hongjie Wang, Baolin Bian, Jian Yang, Xiaolu Wei, Yanyan Zhou, Nan Si, Haiyu Zhao

**Affiliations:** Institute of Chinese Materia Medica, China Academy of Chinese Medical Sciences, Beijing 100700, China; zzjjyyy@163.com (J.Z.); hjwang@icmm.ac.cn (H.W.); blbian@icmm.ac.cn (B.B.); jyang@icmm.ac.cn (J.Y.); xlwei@icmm.ac.cn (X.W.); yyzhou@icmm.ac.cn (Y.Z.)

**Keywords:** aristolochic acid Ι, hepatotoxicity, lipidomics, biomarkers

## Abstract

Aristolochic acid I (AAI) was regarded as the major toxic component of aristolochic acid (AA). In addition to aristolochic acid nephropathy (AAN), liver cancers induced by AAI has aroused increasing attention recently. In this paper, the discovery of diagnostic biomarkers for AAI-induced liver injury has been studied, especially for the lipid markers. From the histopathological characteristics, the injury was observed clearly in the liver apart from the kidney after 30 mg/kg of AAΙ treatment for one week, while the lesion alleviated after AAΙ discontinuance. The serum biochemical indexes were manifested to the normal tendency after AAΙ discontinuance for two weeks. According to the evaluation of pathology slices and serum biochemical indexes, they indicated that the hepatotoxicity induced by AAΙ was reversible to some extent. A total of 44 lipid markers were identified in the liver, as well as 59 in the serum. Twenty-six common lipid markers were observed in both serum and liver. Furthermore, nine out of 26 lipids exhibited the excellent diagnostic ability to differentiate the control group from the AAΙ group and AAΙ discontinuance group with high sensitivity and specificity. The changed lipid markers might serve as characteristics to explain the mechanisms of pathogenesis and progression in hepatotoxicity induced by AAΙ.

## 1. Introduction

Aristolochic acids (AA), a kind of nitrophenanthrene carboxylic acid compounds, were widely distributed in *Aristolochia* and *Asarum* plants of the Aristolochiaceae family. *Aristolochia* was extensively possessed with multi activities for antitumor, antibacterial, anti-inflammatory, analgesic biological activities, and other pharmacological effects. Meanwhile, the increasing emphasis on the toxicity of AA has been laid in recent years. Aristolochic acids I (AAI) was regarded as the major toxic chemical from *Aristolochia*. Aristololactam I (ALI) was the main metabolite of AAI by nitro-reduction reaction in vivo. Both the AAI and ALI could induce the apoptosis of cultured renal epithelial cells through the activation of caspase 3-dependent pathway [1].

AA could induce the apoptosis of proximal tubular cell through various mechanisms, including mitochondrial DNA depletion, respiratory chain defects, and endoplasmic reticulum stress [2,3]. Meanwhile, AA induced the accumulation of methylglyoxal (MGO) and N^ε^-(carboxymethyl) lysine (CML) has become an important and novel pathway in the pathogenic mechanism for aristolochic acid nephropathy (AAN). The serious kidney damage induced by AA was related to an increase and accumulation of MGO and CML. It caused the tubular atrophy with decreased renal function. Meanwhile, creatinine clearance, GSH levels, and the intra-renal antioxidant capacity were descended [4]. Recently, according to the reports [5,6], apart from AAN, AAI and AAI-DNA adduct could induce the liver cancer through characteristic adenine-to-thymine transversions. The results suggested that the herbal medicines containing AA had the potential mutagenic effects in liver. AAI has been listed as one of the major risk factors for liver cancers. Although these results were still controversial, the liver injury caused by AAI was obvious, which attracted increasing attention world-wide. As the continuation of this work, the discovery for the diagnostic biomarkers of AAI-induced liver injury has been considered to be very significant and urgent, especially for lipid markers.

However, in recent years, lipidomics has become a powerful tool for the safety evaluation [7]. It was defined as the completely quantitative determination of lipids in cells, tissues, or biological fluids [8,9]. The researches of lipid homeostasis disorder in liver injury induced by AAI have not been reported. Liver was a major organ for fatty acid oxidation and ketone body formation. The characteristically pathologic changes in the liver were always companied with the disturbance of lipid homeostasis. It has been reported that liver damage could notably affect the normal processes of lipid metabolism through increasing lipid synthesis, reducing the ability of fatty acid β-oxidation, inducing oxidative stress and disturbing thyroid hormone pathways in liver [10]. Thus, the systemic analysis of the disorder in lipid metabolism was a feasible approach for the diagnosis of AAI liver injury.

In the present study, the lipid biomarkers in the AAI treatment group and AAI discontinuance groups were compared to the difference among them. The histopathology of liver and kidney and the biochemical function were evaluated. The injury was identified in liver after the AAI treatment for one week, while the lesion was alleviated after AAI discontinuance for two weeks. The liver exhibited the strong tendency for self-repairing capability. To confirm the potential lipid biomarkers which could be applied in clinical diagnosis, the common lipids in both liver and serum were screened and identified. The disturbances in lipid homeostasis were closely related to the pathogenesis and progression of liver injury.

## 2. Results and Discussion

### 2.1. Tissue Histological Characteristics

**Figure 1:**
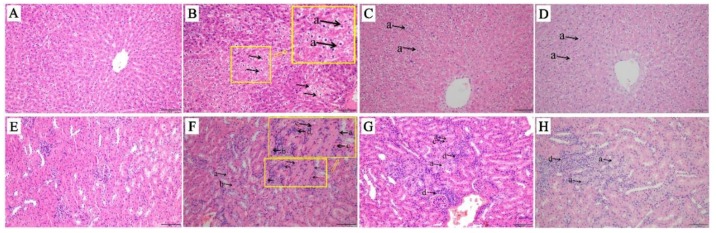
Liver histological structure of rats with 30 mg/kg/d of aristolochic acid I (AAΙ) oral administration for one week and AAΙ discontinuance for one week and two weeks. (**A**) No obviously histopathological changes in the liver of the control group. (**B**) Diffuse swollen (in the yellow box) and vacuolar degeneration in hepatocyte (a→) in the liver of AAΙ group. (**C**) and (**D**) Mild focal hepatocyte swollen and vacuolar degeneration (a→) in the liver of one-week group and two-week group, respectively. (**E**) No obviously histopathological changes in the kidney of control group. (**F**) The epithelial cell of renal tubule in cortex showed multiple focal swollen and degeneration (a→). The acidophily increased in cytoplasm (b→). Necrosis, karyopyknosis, karyolysis, and even disappearance (c→) in individual epithelial cell. Hyperplasia of the mesenchymal cell around the injury renal tubules (d→). (**G**) and (**H**) Multiple focal swollen, degeneration, lumen narrowing, mesenchymal cell hyperplasia in the kidney of one-week group and two-week group, respectively.

The H&E staining of liver and kidney sections were carried out (Figure 1). The histological findings indicated that in the AAΙ group, the liver tissue showed diffuse swelling accompanied with vacuolar degeneration in hepatocyte. In kidney, the renal tubular epithelial cells in cortex showed the multiple focal swollen. Meanwhile, the degeneration of renal tubular epithelial cells was also observed. The acidophil appeared to increase in the cytoplasm of kidney. In general, the severity of the lesion in liver was milder than that in kidney obviously. The kidney was the main impairment target organ in the AAΙ-induced injury. In the discontinuance groups, the mild focal hepatocyte swelling and vacuolar degeneration were observed in liver but with no significant difference. The degree of pathological changes was lightened compared to the AAΙ group. In kidney, multiple focal swelling, degeneration, lumen narrowing, and mesenchymal cell hyperplasia were observed. The lesion was alleviated during the kidney repair process.

### 2.2. Serum Biochemical Index Results

A total of 11 serum biochemical indexes related to the function of liver and kidney were detected, including ALT, AST, TBA, ALB, GLB, LDH, CHE, TP, Crea, BUN, and UA. Compared with the control group, the concentration of ALB, TP, and CHE were significantly decreased in the AAΙ group. Discontinuance of AAΙ for two weeks later, ALT, AST, TP, TBA, ALB, GLB, LDH, and CHE were exhibited the tendency to the normal level (Table 1).

AST and ALT, the liver function enzymes, could commonly assess the severity of hepatic disease [11]. Compared to the AAΙ group, ALT displayed a more than twofold increase in the one-week group. The concentration of ALT almost returned to the normal level in the subsequent two-week group. In this study, AST/ALT ratio was higher than 1.2 in the AAΙ group. Specifically, the ratio of AST/ALT >1 was a significant predictor of liver dysfunction [12,13]. CHE was obviously declined in AAΙ group compared to the control group. Due to the concentration of CHE in serum decrease for the liver presented symptoms of damage, CHE could not be timely supplemented in serum [14]. While compared to the AAΙ group, CHE remarkably increased 30% and 40% in the discontinuance groups, respectively. Meanwhile, compared to the control group, LDH and Crea were significantly increased in the AAΙ group, which suggested the impairment of the function of glomerular filtration.

The biochemical test items could effectively reflect the anabolic status and the degree of hepatocyte damage, so as to provide the referenced basis for the treatment and prognosis of patients with liver diseases [15]. The data showed that the disturbance in the biochemical indexes in different levels was closely related to the liver function. Interestingly, in the discontinuance groups, the majority of the aforementioned indexes presented the trend to the normal level. The liver exhibited the strong tendency of self-repairing capability.

### 2.3. Examination of Concentration of AAΙ and ALΙ in Liver and Serum

The precursor and product ions of multiple reaction monitoring (MRM) detection were listed (Table 2). The limit of quantitation (LOQ) for AAΙ and ALΙ was 5 ng/mL based on S/N ≥ 10. The method offered a good linearity. The r value was above 0.99 (Table 3 and Table 4). The concentrations of AAΙ and ALΙ in liver and serum in the AAΙ group were shown in Table 5, except for the concentration of ALΙ in the serum since it was below the LOQ. In addition, the concentrations of AAΙ and ALΙ in liver and serum were undetectable in the discontinuance groups.

### 2.4. Identification of the Potential Lipid Biomarkers

**Figure 2:**
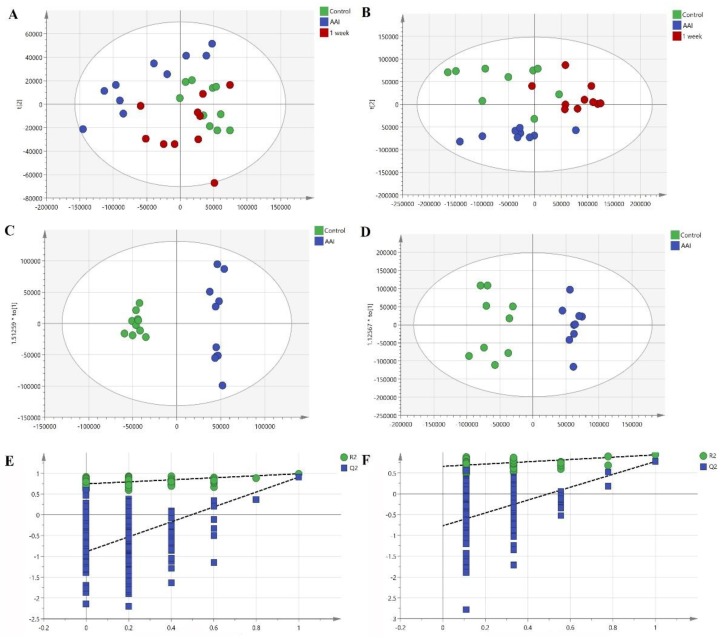
Multivariate statistical analysis of control group, AAΙ group, one-week group in serum and liver samples. (**A**) principal components analysis (PCA) scores plot of three groups in serum. (**B**) PCA scores plot of three groups in liver. (**C**) orthogonal projections to latent structures discriminant analysis (OPLS-DA) scores scatter plot in serum. (**D**) OPLS-DA scores scatter plot in liver. (**E**) OPLS-DA validation plot in serum. (**F**) OPLS-DA validation plot in liver.

From the original MS data obtained from liver and serum samples, the multivariate statistics, unsupervised model of principal components analysis (PCA), and supervised model of orthogonal projections to latent structures discriminant analysis (OPLS-DA) were applied to gain an overview of the lipidomic profilings in all groups and set an obvious separation of the control group and AAΙ group respectively, with high statistical values of R^2^ and Q^2^. In the PCA score, the cumulative R^2^X and Q^2^ were 0.802, 0.651 in serum, 0.865, and 0.727 in liver. In the OPLS-DA model, R^2^X, R^2^Y, and Q^2^ were 0.865, 0.976, and 0.886 in serum, 0.725, 0.936, and 0.777 in liver. No over-fitting was observed according to the permutation (Figure 2).

The comparisons of difference among the three groups (control group, AAΙ group, one-week group) in liver and serum were then performed based on the parameters. In the AAΙ group, a total of 59 lipid biomarkers including 36 phosphatidylcholines (PCs), 13 sphingolipids (SMs), five phosphatidylethanolamines (PEs), four glucosylceramides (GlcCers), and one ceramide (Cer) were significantly altered in serum. Fourty-four lipid biomarkers including 22 PCs, 10 PEs, 7 SMs, 3 Cers, and 2 GlcCers were remarkably identified in liver.

**Figure 3:**
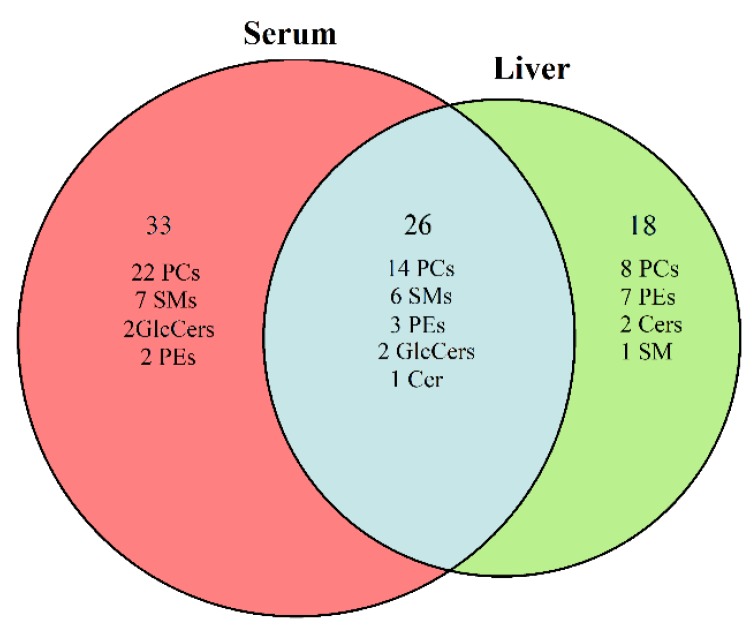
Venn diagram. Each circle represented the potential lipid markers based on the comparisons of the AAΙ group versus the control group in serum and liver, respectively.

**Figure 4:**
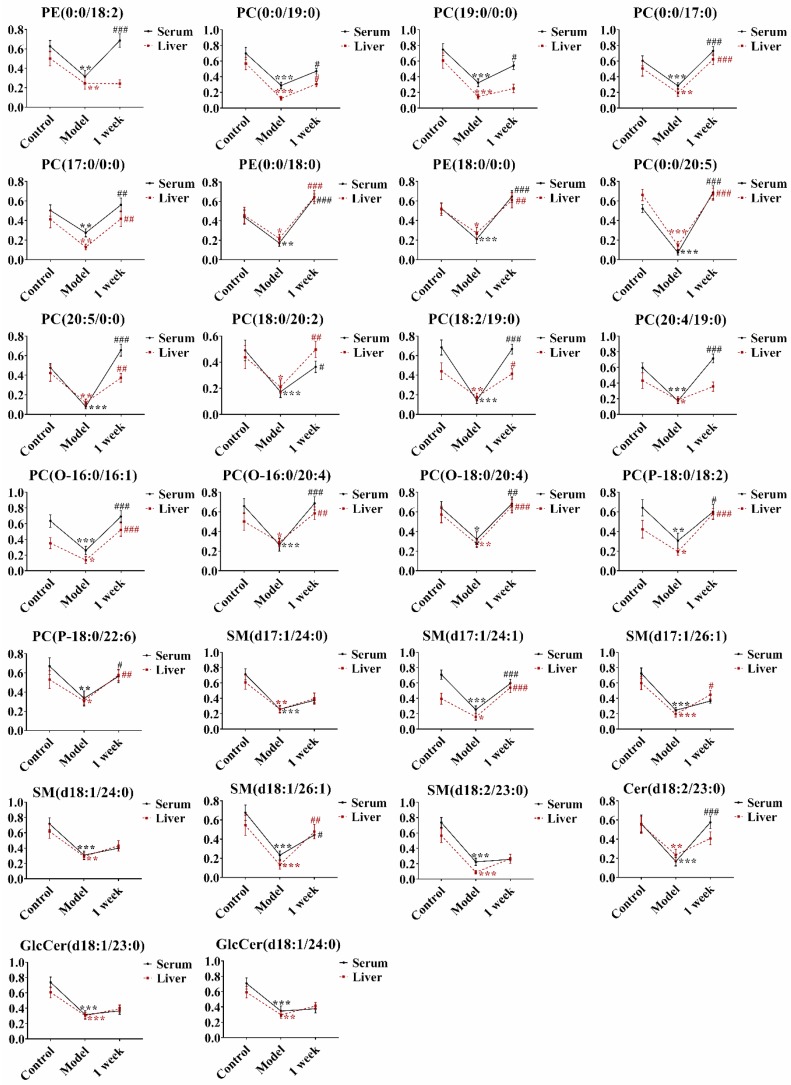
The tendency of level variation of significantly differentially expressed lipids in both liver and serum in the control group, AAΙ group, and one-week group. * *P* < 0.05, ** *P* < 0.01, and *** *P* < 0.001, the control group compared to the AAΙ group. ^#^ *P* < 0.05, ^##^ *P* < 0.01, and ^###^ *P* < 0.001, the one-week group compared to the control group.

Taken together, as shown in the Venn diagram (Figure 3), compared with the control group, 26 common lipid markers were observed in both serum and liver, which illustrated a close relationship between the hepar and blood in many aspects and the circulating serum lipid profile might reflect the status of the liver function. The data were shown in Table S1 and Table S2. Additionally, in the AAΙ group, 33 lipids were altered in serum individually (Table S3). Eighteen lipids were identified in liver as well (Table S4). The lipids changed levels among the three groups werein difference, but the tendency of these variations was consistent (Figure 4). For instance, compared with the control group, the level of PE (0:0/18:2) were descended significantly in both serum and liver in AAΙ group. Then, the level returned obviously in serum, whereas it exhibited the increased tendency with no significance in liver of the one-week group. Compared with control group, the level of PE (0:0/18:0) in AAΙ group was significantly decreased in both liver and serum samples, and ascended remarkably in the one-week group compared with AAΙ group.

**Figure 5:**
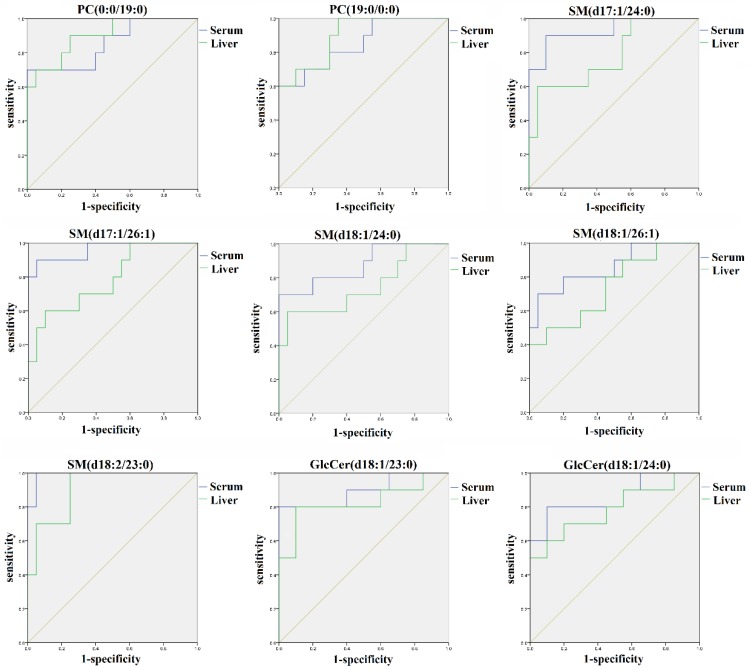
Receiver operating characteristic (ROC) curves of nine out of 26 common lipids from the control group, AAΙ group, and one-week group based on OPLS-DA analysis. The associated AUC, specificity, and sensitivity values of five SMs, two PCs, and two GlcCers were exhibited.

To evaluate the lipid markers’ discriminative validity, the receiver operating characteristic (ROC) curve analysis was further performed in the validation set. The results indicated that nine out of 26 identified lipid markers including PC (0:0/19:0), PC (19:0/0:0), SM (d18:2/23:0), SM (d17:1/24:0), SM (d18:1/24:0), SM (d17:1/26:1), SM (d18:1/26:1), GlcCer (d18:1/23:0), and GlcCer (d18:1/24:0) exhibited excellent diagnostic abilities to differentiate between the control group and the AAΙ group. As shown in Figure 5, nine lipid markers in both liver and serum had the high sensitivity and specificity with the area under the curve (AUC) of 0.740 or larger. Therefore, they might be the suitable metabolites for the detection of AAΙ hepatotoxicity. Specifically, SM (d18:2/23:0) exhibited the distinguished ability with an AUC value of 0.990, a specificity of 95.0% in serum, as well as an AUC value of 0.910, a specificity of 75.0% in liver.

Changes in the lipid markers were closely associated with histopathological and biochemical abnormalities of liver. The underlying mechanism responsible for the observed alterations of multiple lipid species in the liver and serum of rats with AAΙ is presently unclear. Phospholipid metabolism has been proven to be critically associated with the liver regeneration [16]. A number of previous studies have reported the relation between the abnormal phospholipids and the liver failure [17,18]. In the lipid metabolism network, phospholipids containing one of the 20:4 fatty acid chain were the reservoir of a series of biologically active lipid mediators. It has been reported that patients with steatohepatitis had less hepatic arachidonic acid (20:4 n-6) than healthy people [19]. In this study, as the main source of arachidonic acid, PCs were converted into it under the inflammatory condition, which caused the metabolic disturbance of inflammatory mediators and the levels of phospholipids were decreased in the AAΙ group. The previous research also supported the results [20]. In addition, pretreatment with PC could significantly prevent the increases of serum ALT and AST, and reduce the reactive oxygen species levels [21]. This might illustrate that the level of ALT and AST were reversed back to the normal tendency after discontinuance of AAΙ for two weeks.

SMs were a source of bioactive metabolites to regulate many cellular processes [22]. Deregulation of SM synthesis and transport were associated with a variety of metabolic disorders [23]. Previous studies have been confirmed that SMs and their metabolism played significant roles in regulating the hepatic ischemia, ischemia/reperfusion (I/R) injury, drug-induced injury such as the acetaminophen (APAP) toxicity, and the liver regeneration. They had a great impact on the therapeutic development of diverse liver diseases and might serve as the prognostic and diagnostic markers [24,25]. Cers were mainly synthesized via de novo synthesis and also produced by degradation of sphingomyelin or cerebrosides [26,27]. A series of studies have reported that the levels of LDL, HDL, and VLDL cholesterol in serum exhibited a tendency to decline in patients with liver decompensation [28,29]. SMs and Cers were all involved in VLDL, LDL, and HDL. It was assumed that SMs and Cers lipid species would also be reduced in liver injury [28,30,31].

GlcCers, a type of SMs, were generated by glucosylceramide synthase (GCS). GCS could vary the hepatic lipid metabolism and improve glucose tolerance [32,33]. Mammalian GlcCers mainly contain sphingosine (d18:1). The changes of GlcCer (d18:1/23:0) and GlcCer (d18:1/24:0) were both decreased in the AAΙ group. The inhibition of GCS was able to trigger the proliferation or apoptosis of hepatic cells and cascadedly lead to the decrease of GlcCer levels. Then, the exerted pro-mitogenic effects happened, which contributed to the process of decreased activity of hepatocytes [34,35,36]. Additionally, it has been reported that GluCer (d18:1/24:0) could reflect the severity of drug-induced hepatic phospholipidosis in hepatocytes and would be a potential blood biomarker for the diagnosis.

Changes of the lipid markers were closely associated with toxicity. In addition to the hepatotoxicity, PC (18:3/0:0) has been reported to be the suitable metabolic marker with the high sensitivity and specificity for early nephrotoxicity prior to the conventional biochemical or histological abnormalities [37]. Moreover, the plasma lipidomic analysis in humans manifested the relationship of PC species such as PC (38:3), PC (18:2/0:0) et al. with risk measures of metabolic diseases [38,39,40]. Long chain SMs such as SM (d18:1/24:1) has been found to be downregulated in hepatitis patients compared with in healthy controls [41]. The study demonstrated that the abnormal lipid metabolism was associated with the increased expression of COX-2, ROS, and NF-κB activation, as well as the inflammatory cell infiltration and fatty degeneration in the chronic kidney disease of mice [42].

In general, the association between the altered lipidomic profilings and AAΙ hepatotoxicity was explored. The results manifested that the hepatic and serous lipid metabolism had the remarkable correlation with liver function. The perturbations of the lipids homeostasis could be partly reversed after the discontinuance of AAΙ for one week. This was corresponding to the histological evaluation that the hepatic lesion began to be lightened gradually after AAΙ discontinuance. The identified lipid biomarkers provided a possibility for diagnosing AAΙ liver injury in an early detection.

## 3. Materials and Methods

### 3.1. Chemicals and Reagents

Aristolochic acid Ι (AAΙ) and aristololactam Ι (ALΙ) were obtained from Saibaicao Technology Co., Ltd. (Beijing, China). AAΙ was suspended in 0.1% sodium carboxymethylcellulose (CMC-Na) solution. Buspirone was purchased from the National Institutes for Food and Drug Control of China (Beijing, China). Methanol and acetonitrile of mass spectrometry (MS) grade were obtained from the Thermo Fisher Company (Fair Lawn, NJ, USA). Formic acid was purchased from Sigma Chemical Co., Ltd. (ST Louis, MO, USA). The purities of all the standard references were over 98%. All other chemicals and solvents were of analytical grade.

### 3.2. Animals and Sample Collection

Male SD rats, weighting 260 ± 20 g, were purchased from the Beijing Animals Science Biotechnology Co., Ltd. (Beijing, China). After one week of adaptive feeding, the rats were randomly divided into four groups: Control group, AAΙ oral-administration group (AAΙ group), discontinuance of AAΙ for one-week group (one week group), and discontinuance of AAΙ for two- week group (two week group), with 10 rats in each group. The AAΙ group was orally administrated 30 mg/kg body weight/ day of AAΙ. All of the hepatic and renal tissues were flash-frozen in liquid nitrogen after washing with normal saline, and were stored at −80 °C until analysis. All serum samples were centrifuged at 3000 rpm, 4 °C for 10 min and the supernatant was collected and stored at −80 °C freezer until use.

### 3.3. Biochemical Analysis

The levels of serum biochemistry including creatinine (Crea), alanine aminotransferase (ALT), aspartate aminotransferase (AST), blood urea nitrogen (BUN), uric acid (UA), lactic dehydrogenase (LDH), total protein (TP), albumin (ALB), globulin (GLB), total bile acid (TBA), and cholinesterase (CHE) were detected by the Olympus AU480 automated biochemistry analyzer (Olympus, Tokyo, Japan).

### 3.4. Histological Evaluation

Liver and kidney tissues were fixed by 4% paraformaldehyde solution, dehydrated, embedded in paraffin, prepared at 4 μm thickness, and stained with hematoxylin and eosin (H&E), then observed and described in a panoramic scanner for histopathological examination.

### 3.5. Sample Preparation for Lipidomics

#### 3.5.1. Treatment of Serum Samples

The serum samples were thawed at room temperature. 600 μL chloroform/methanol (3:1) was added to an aliquot of 100 μL serum and sonicated for 3 min. Then, 100 μL water was added for vortex-mixing, and centrifuging at 12,000 rpm, 4 °C for 10 min. 300 μL of the subnatant chloroform was collected to concentrate under the stream of nitrogen, adding 400 μL isopropanol /acetonitrile (1:1) to redissolve, then sonicated and centrifugated at 12,000 rpm, 4 °C for 10 min again. The supernatant was finally analyzed by UPLC-Q-Exactive MS.

#### 3.5.2. Treatment of Liver Samples

The liver samples were thawed at room temperature, homogenated, and added 1 mL chloroform/methanol (3:1), then sonicated for 1 h. The mixture was added 100 μL water for vortex-mixing, centrifuging at 13,200 rpm, 4 °C for 10 min. Then, 500 μL of the subnatant chloroform was concentrated under the stream of nitrogen. Adding 300 μL isopropanol/acetonitrile (1:1) to the dried samples, vortex-mixing for 40 s and centrifuging at 12,000 rpm, 4 °C for 5 min, finally the supernatant was taken for analysis.

### 3.6. Quantitative Analysis of AAI and ALI in Liver and Serum

#### 3.6.1. Preparation of Stock Solutions, Calibration Standards, and Internal Standard Solutions

For the preparation of calibration curves, standard stock solutions contained AAΙ and ALΙ were prepared in the methanol at the concentration of 1.0 mg/mL. Working standard solutions were prepared by serial dilution of the stock solutions with the concentrations of 10,000, 8000, 5000, 2000, 1000, 500, 200, 100, and 50 ng/mL to create the necessary concentrations. Buspirone as internal standard (IS) was prepared at 25 ng/mL in acetonitrile/methanol (1:1). All solutions were stored at 4 °C for further analysis.

#### 3.6.2. Preparation for the Serum and Liver Samples

Aliquots of 50 μL serum or homogenated extraction of liver samples were accurately taken and 5 μL methanol was added, then spiked with 300 μL internal standard solution. After the sufficient vortex for 3 min, 4000 rpm, 3 min centrifugation was performed at 4 °C for 15 min. Finally, the supernatant was taken for the LC-MS/MS analysis.

#### 3.6.3. UPLC-MS/MS Conditions

Agilent 1200 HPLC (Agilent Technologies, Santa Clara, CA, USA) was used. Kinetex-C 18 110A column (3 × 30 mm, 2.6 μm, Phenomenex) was operated at 30 °C and the flow rate was set at 0.8 mL/min for separation of AAΙ and ALΙ. The mobile phase consisted of (A) water containing of 10 mmol/L ammonium acetate and (B) acetonitrile. Gradient conditions were as follows: 0–0.5 min, 10% B; 0.5–0.8 min, 10–95% B; 0.8–2.5 min, 95% B. A 10 μL aliquot of each sample was injected. All samples were kept at 4 °C throughout the analysis.

MS was performed on an API 4000 Qtrap system (Applied Biosystems, Foster City, CA, USA). Electrospray ionization (ESI) was performed in the positive ion mode. Curtain gas (CUR), nebulizer gas (GS1), and turbo-gas (GS2) were set at 15 psi, 60 psi, and 60 psi, respectively. The ionspray voltage was 5.0 kV, and the temperature was 550 °C. Nitrogen was employed as the collision gas. AAΙ and ALΙ were analyzed using the scheduled MRM. Data acquisitions were performed using the Analyst 1.5.2 software (Applied Biosystems). Multiquant software (Applied Biosystems) was used to quantify AAΙ and ALΙ.

#### 3.6.4. Analytical Validation

A typical standard curve was prepared by adding the internal standard to serial dilution of the stock solution. Linear regression analysis obtained from the calibration curve was used to calculate the corresponding AAΙ and ALΙ in samples. LOQ was determined from the standard solution with the concentration that resulted in a peak with a signal to noise ratio (S/N) greater than 10:1.

### 3.7. UPLC/Q-Exacitve/MS Analysis for Lipidomics

The UltiMate™ 3000 Rapid Separation LC (RSLC) system (Thermo Scientific, USA) was performed for the relative quantification of various species of lipids. For C18 separation, the binary gradient program consisted of acetonitrile/water (60/40) (mobile phase A) and isopropanol/ acetonitrile (90/10) (mobile phase B). Both A and B contained 0.1% formic acid and 10 mmol/L ammonium acetate. All the samples were eluted using the following linear gradient conditions: 0–2 min, 20–30% B; 2–5 min, 30–45% B; 5–6.5 min, 45–60% B; 6.5–12 min, 60–65% B; 12–14 min, 65–85% B; 14–17.5 min, 85–100% B; 17.5–18 min, 100–100% B, and the equilibration time was 1.5 min with 20% B. The Waters Acquity UPLC HSS T3 column (2.1 × 100 mm, 1.8 µm) was operated at 50 °C and the flow rate was set at 0.3 mL/min for separation of lipids.

A Thermo Scientific™ Q Exactive hybrid quadrupole Orbitrap mass spectrometer equipped with a HESI-II probe was performed in a positive ionization mode. The HESI-II spray voltages was 3.7 kV. The heated capillary temperature was 320 °C. The heated vaporizer temperature was 300 °C. The sheath gas pressure was 30 psi. The auxiliary gas setting was 10 psi and the collision gas was at a pressure of 1.5 mTorr. The parameters of the full mass scan were resolution of 70,000, auto gain control target under 1.0 × 106, maximum isolation time of 50 ms, and the scanning range of *m/z* was 50–1500 Da.

### 3.8. Data processing and Statistical Analysis

All of the LC-MS data were processed by the Progenesis QI software (Nonlinear Dynamics, Newcastle, UK) for peak alignment and peak extraction, providing chromatogram retention time, m/z, and characteristic peak strength information for further statistical analysis.

Quality control (QC) samples were prepared by mixing equal aliquots of all the samples. The cluster of the QC samples in the PCA scores scatter plot exhibited a satisfactory stability and repeatability of the lipidomic analysis approach. PCA scores plot has been added in Figure S1. In this experiment, five blank samples and following three QC samples were used first to balance the column conditions. Then, one QC sample was inserted every 6–8 samples for monitoring the stability and repeatability of the whole liquid quality system. The multivariate analysis was performed by SIMCA14.1 (Umetrics AB, Umea, Sweden) including principal component analysis (PCA) and orthogonal partial least squares discriminant analysis (OPLS-DA). Based on the criteria of variable importance in the projection (VIP) values (VIP > 1) obtained from the OPLS-DA model, t-test (*P* < 0.05) and fold change values (FC > 1.5 or FC < 0.7) was set as the indexes for screening the potential biomarkers with significant difference. Then, using the Skyline software for the relative quantification of the selected potential lipid structures through multistage mass spectra information fragments. In the receiver operating characteristic (ROC) curve analysis, the diagnostic index of sensitivity and specificity of lipids in serum and liver tissue was calculated by the SPSS 22.0 software (IBM Crop., Armonk, NY, USA). Youden index = sensitivity − (1 − specificity). The maximum of Youden index was defined as the cutoff value of the lipid metabolism.

All data were presented as means ± standard error of the mean (SEM). Statistical significance between the different groups was determined by one-way ANOVA followed by least significant difference (LSD) post hoc tests using GraphPad prism version 7.01 (GraphPad Software, Inc. Version 7.01). Statistically significant level was set at *P* < 0.05.

## 4. Conclusions

In the present study, the kidney was the main impaired target organ for AA-induced injury compared with liver damage. Along with the AAΙ discontinuance, the lesion severity was alleviated. The concentrations of AAΙ and ALΙ in liver and serum could not be detected in the discontinuance groups according to the LC-MS/MS quantitative analysis. A total of 44 lipid markers in liver, as well as 59 in serum were identified. Twenty-six common lipids were observed in both serum and liver. According to the evaluation of histological characteristics, serum biochemical indexes, and the lipidomic profilings, it indicated that the hepatotoxicity induced by AAΙ could be reversible to some extent. Furthermore, the hypothesis that the hepatocyte damage would be related to the liver cancer needs further research in the future.

## Figures and Tables

**Table 1:** Effect of AAΙ on serum biochemical indexes in four groups.

**Group**	**ALT (U/L)**	**AST (U/L)**	**TBA (μmol/L)**	**ALB (g/L)**	**GLB (g/L)**	**CHE (U/L)**
Control	52 ± 6.07	93.25 ± 25.47	28.22 ± 17.41	31.53 ± 0.89	20.60 ± 0.48	102.75 ± 19.48
AA	64.14 ± 24.39	112.2 ± 34.19	30.16 ± 12.99	29.63 ± 0.69 ^###^	19.87 ± 1.76	84.44 ± 17.30 ^#^
1 week	100.63 ± 7.58 ***	132.88 ± 27.05	35.77 ± 18.06	30.29 ± 1.27	18.82 ± 1.15	107.10 ± 17.80 **
2 week	55.40 ± 7.04	107.22 ± 14.13	20.98 ± 11.88	31.71 ± 0.66 ***	19.45 ± 0.90	117.00 ± 15.40 ***
**Group**	**TP (g/L)**	**LDH (U/L)**	**Crea (μmol/L)**	**BUN (mmol/L)**	**UA (μmol/L)**	
Control	52.43 ± 1.71	367.71 ± 127.57	15.78 ± 1.72	4.82 ± 0.45	27.63 ± 8.28	
AA	48.54 ± 1.42 ^###^	643.00 ± 315.26 ^##^	19.80 ± 6.03 ^#^	5.19 ± 0.94	28.17 ± 19.16	
1 week	49.05 ± 1.60	349.89 ± 133.01 **	21.50 ± 6.09	5.37 ± 0.63	15.89 ± 7.72	
2 week	51.46 ± 1.82 ***	305.67 ± 96.07 **	22.33 ± 1.75	5.54 ± 0.78	14.00 ± 4.12*	

Compared with control group: **^#^** *P* < 0.05, **^##^** *P* < 0.01, **^###^** *P* < 0.001. Compared with AA-oral administration group: * *P* < 0.05, ** *P* < 0.01, *** *P* < 0.001.

**Table 2:** Optimized multiple reaction monitoring (MRM) pairs and parameters for AAΙ and aristololactam I (ALΙ).

Analyte	*m/z*		DP (V)	CE (V)
	Precursor	Product		
AAΙ	359.1	298.8	66	15
ALΙ	294.1	279.2	146	39
Buspirone (IS)	386.2	122.2	91	49

**Table 3:** Linearity, limit of quantitation (LOQ) of the LC-MS/MS assay for AAΙ and ALΙ in serum.

Analyte	Equation	Correlation Coefficient (r)	Linear Range (ng/mL)	LOQ (ng/mL)
AAΙ	y = 0.000512 x + 0.000337	0.9954	5–1000	5
ALΙ	y = 0.000425 x − 0.000135	0.9928	5–1000	5

**Table 4:** Linearity, LOQ of the LC-MS/MS assay for AAΙ and ALΙ in liver.

Analyte	Equation	Correlation Coefficient (r)	Linear Range (ng/mL)	LOQ (ng/mL)
AAΙ	y = 0.000403x + 0.000775	0.9993	5–1000	5
ALΙ	y = 0.00045 x + 0.000265	0.9937	5–1000	5

**Table 5:** The level of AAΙ and ALΙ (ng/mL) in serum and liver in the AA group (*n* = 6).

**Analyte**	**Serum**	**Serum**	**Serum**	**Serum**	**Serum**	**Serum**	**Mean ± SEM**
AAΙ	75.1	67.6	65.5	81.5	55.5	52.4	66.27 ± 4.55
ALΙ	-	-	-	-	-	-	-
**Analyte**	**Liver**	**Liver**	**Liver**	**Liver**	**Liver**	**Liver**	**Mean ± SEM**
AAΙ	98.6	50.2	97.0	95.8	62.5	79.4	80.58 ± 8.32
ALΙ	42.7	61.2	32.5	37.4	86.4	68.3	54.75 ± 8.50

-: the concentration of the analyte was below the LOQ.

## Supplementary Materials

The following are available online. Table S1: Identification of 26 common lipid markers in serum of AAΙ group and control group; Table S2: Identification of 26 common lipid markers in liver of AAΙ group and control group; Table S3: Identification of 33 lipid markers in serum of AAΙ group and control group; Table S4: Identification of 18 lipid markers in liver of AAΙ group and control group; Figure S1: (**A**) The PCA scores plot of QC and on-test samples in serum. (**B**) The PCA scores plot of QC and on-test samples in liver.
