# Supplementary material for: The Disturbance of Hepatic and Serous Lipids in Aristolochic Acid Ι Induced Rats for Hepatotoxicity Using Lipidomics Approach
Source: Molecules. 2019 Oct 17;24(20):3745. doi: 10.3390/molecules24203745 (PMC6832582; doi:10.3390/molecules24203745)
Supplement: Supplementary file 1 [file molecules-24-03745-s001.pdf]

Supplementary Materials

Table S1. Identification of 26 common lipid markers in serum of AAI group and control group

lipid markers	m/z	VIP	*P*	Fold change (AAI/Control)	Fold change (AAI/1 week)
PE(0:0/18:2)	478.2928	1.2900	0.0017	0.5029	0.4595
PE(0:0/18:0)	482.3241	1.2138	0.0035	0.3895	0.2677
PE(18:0/0:0)	482.3241	1.3387	0.0002	0.4031	0.3276
PC(0:0/17:0)	510.3554	1.3657	0.0006	0.4716	0.3897
PC(17:0/0:0)	510.3554	1.0711	0.0046	0.5463	0.4900
PC(0:0/19:0)	538.3867	1.5354	0.0002	0.4138	0.6136
PC(19:0/0:0)	538.3867	1.5509	0.0002	0.4320	0.5963
PC(0:0/20:5)	542.3241	1.7496	0.0000	0.1395	0.1058
PC(20:5/0:0)	542.3241	1.6319	0.0000	0.1683	0.1221
Cer(d18:2/23:0)	634.6133	1.4016	0.0010	0.2966	0.2901
PC(O-16:0/16:1)	718.5745	1.3913	0.0009	0.4071	0.3741
PC(O-16:0/20:4)	766.5745	1.4033	0.0008	0.4023	0.3858
PC(P-18:0/18:2)	770.6058	1.1657	0.0091	0.4789	0.5105
PC(O-18:0/20:4)	796.6215	1.1759	0.0057	0.5038	0.4705
GlcCer(d18:1/23:0)	798.6817	1.5428	0.0001	0.4288	0.8654
SM(d17:1/24:1)	799.6687	1.6417	0.0000	0.3578	0.4266
SM(d18:2/23:0)	799.6687	1.7780	0.0000	0.3034	0.8707
PC(18:2/19:0)	800.6164	1.8735	0.0000	0.2102	0.2156
SM(d17:1/24:0)	801.6844	1.6496	0.0000	0.3622	0.6875
GlcCer(d18:1/24:0)	812.6974	1.3018	0.0018	0.4899	0.9215
PC(18:0/20:2)	814.6320	1.2318	0.0026	0.3526	0.4743
SM(d18:1/24:0)	815.7001	1.4664	0.0004	0.4297	0.7708
PC(P-18:0/22:6)	818.6058	1.1907	0.0095	0.4980	0.5911
PC(20:4/19:0)	824.6164	1.6120	0.0000	0.2887	0.2403
SM(d17:1/26:1)	827.7001	1.6887	0.0000	0.3384	0.6722
SM(d18:1/26:1)	841.7157	1.5429	0.0001	0.3442	0.5246

Table S2. Identification of 26 common lipid markers in liver of AAI group and control group

lipid markers	m/z	VIP	*P*	Fold change (AAI/Control)	Fold change (AAI/1 week)
PE(0:0/18:2)	478.2928	1.3766	0.0164	0.4895	1.0136
PE(0:0/18:0)	482.3241	1.0871	0.0226	0.4784	0.3392
PE(18:0/0:0)	482.3241	1.1151	0.0118	0.5318	0.4521
PC(0:0/17:0)	510.3554	1.4290	0.0084	0.3845	0.3116
PC(17:0/0:0)	510.3554	1.4323	0.0067	0.3127	0.3090
PC(0:0/19:0)	538.3867	2.1675	0.0000	0.2156	0.4009

PC(19:0/0:0)	538.3867	2.0899	0.0002	0.2385	0.5796
PC(0:0/20:5)	542.3241	2.3037	0.0000	0.2191	0.2161
PC(20:5/0:0)	542.3241	1.4656	0.0036	0.2882	0.3256
Cer(d18:2/23:0)	634.6133	1.5946	0.0089	0.4274	0.5781
PC(O-16:0/16:1)	718.5745	1.2183	0.0161	0.3871	0.2604
PC(O-16:0/20:4)	766.5745	1.0897	0.0500	0.5828	0.4995
PC(P-18:0/18:2)	770.6058	1.2144	0.0283	0.4605	0.3363
PC(O-18:0/20:4)	796.6215	1.5443	0.0053	0.4836	0.4207
GlcCer(d18:1/23:0)	798.6817	1.6533	0.0023	0.4990	0.7702
SM(d17:1/24:1)	799.6687	1.3902	0.0107	0.3971	0.2908
SM(d18:2/23:0)	799.6687	2.2485	0.0001	0.1557	0.3343
PC(18:2/19:0)	800.6164	1.4455	0.0126	0.4055	0.4328
SM(d17:1/24:0)	801.6844	1.7671	0.0038	0.4232	0.6477
GlcCer(d18:1/24:0)	812.6974	1.6298	0.0036	0.5030	0.7209
PC(18:0/20:2)	814.6320	1.2621	0.0414	0.4822	0.4252
SM(d18:1/24:0)	815.7001	1.6793	0.0047	0.4738	0.6808
PC(P-18:0/22:6)	818.6058	1.1363	0.0414	0.5812	0.5336
PC(20:4/19:0)	824.6164	1.3587	0.0344	0.4233	0.5138
SM(d17:1/26:1)	827.7001	1.9953	0.0004	0.3232	0.4356
SM(d18:1/26:1)	841.7157	2.0095	0.0019	0.2407	0.2747
